# Phosphatase Inhibition Attenuates the Negative Inotropic Effect of Muscarinic Receptors in the Presence of Serotonin

**DOI:** 10.3390/cimb48070698

**Published:** 2026-07-09

**Authors:** Rebecca Schwarz, Britt Hofmann, Ulrich Gergs, Uwe Kirchhefer, Joachim Neumann

**Affiliations:** 1 Institute for Pharmacology and Toxicology, Medical Faculty, Martin-Luther-University Halle-Wittenberg, Magdeburger Straße 4, D-06097 Halle, Germany; rebecca.schwarz@medizin.uni-halle.de (R.S.); ulrich.gergs@medizin.uni-halle.de (U.G.); 2 Cardiac Surgery, Medical Faculty, Martin-Luther-University Halle-Wittenberg, Ernst-Grube Straße 40, D-06097 Halle, Germany; britt.hofmann@uk-halle.de; 3 Institute for Pharmacology and Toxicology, Medical Faculty, University Münster, Domagkstraße 12, D-48149 Münster, Germany; kirchhef@uni-muenster.de

**Keywords:** isoprenaline, histamine, serotonin, carbachol, cantharidin, human atrium

## Abstract

Cantharidin (CANT) and sodium fluoride inhibit serine/threonine phosphatase (PP) 1 and PP2A in the heart. We hypothesized that Cant and sodium fluoride could weaken the reduction in force by carbachol in the presence of serotonin in human right atrial preparations (HAP). We measured contractility in HAP, and for comparison, in left atria from wild type mice (CD1, WT), mice with human 5-HT_4_-serotonin receptor cardiac overexpression (5-HT_4_-TG), or in mice with human H_2_-histamine receptor cardiac overexpression (H_2_-TG). Isoprenaline (1 µM), histamine (1 µM), and serotonin (1 µM) raised contraction in HAP. Likewise, isoprenaline (1 µM), histamine (1 µM), and serotonin (1 µM) raised contractility in the left atria of WT, H_2_-TG, and 5-HT_4_-TG. Carbachol (1 µM), a muscarinic cholinoceptor agonist, diminished the force of contraction in a time-dependent manner after prior stimulation of force by serotonin, isoprenaline, or histamine in HAP but also in left atria from 5-HT_4_-TG, WT, and H_2_-TG. These anti-β-adrenergic, anti-serotoninergic and anti-histaminergic effects of carbachol on force of contraction were attenuated by 100 µM or 30 µM cantharidin or 3 mM sodium fluoride in HAP and in left atrial preparations from WT, H_2_-TG, and 5-HT_4_-TG, respectively. We conclude that muscarinic cholinoceptor activation may exert a negative inotropic effect in the HAP by stimulating PP1 and/or PP2A, which had previously been inhibited by isoprenaline or histamine or serotonin.

## 1. Introduction

**Figure 1:**
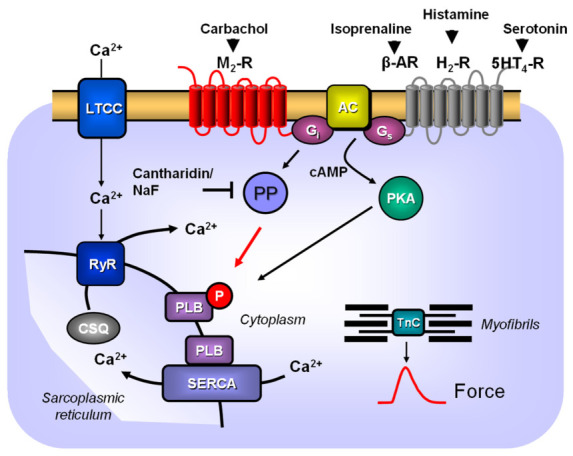
Scheme of putative effects of carbachol in the heart: Ca^2+^ entering the cardiomyocyte through the L-type Ca^2+^ channel (LTCC) triggers in large part the subsequent release of Ca^2+^ from the sarcoplasmic reticulum (SR) through ryanodine receptors (RYR) in the junctional SR. Cardiac relaxation facilitated by an augmented phosphorylation state of phospholamban (PLB) in the free SR and of inhibitory subunit of troponin (TnI) in the myofilaments. PLB inhibits the activity of the SR-Ca^2+^-ATPase (SERCA). When PLB is phosphorylated Ca^2+^ is pumped by SERCA from cytosol into the SR. Protein phosphatases (PP), mainly PP1 and PP2A, can dephosphorylate PLB, TnI, and the LTCC. PP1 and more potently PP2A are inhibited by cantharidin. Carbachol (CAR) stimulates cardiac M_2_-muscarinic receptors (M_2_-R). Carbachol may reduce activity of the adenylyl cyclases (ACs) via pertussis-toxin sensitive inhibitory guanosine-triphosphate binding-proteins (G_i_). In the human atrium, isoprenaline stimulates β-adrenoceptors, while histamine activates H_2_-histamine receptors and serotonin activates 5-HT_4_-serotonin receptors. These three receptors lead, via stimulatory G-proteins (Gs), to augmentation of the activity of adenylyl cyclases (ACs). As a result, the cAMP levels are elevated. Thereafter, the cAMP activates the cAMP-dependent protein kinase (PKA). PKA phosphorylates and stimulates many other proteins as well as PLB.

In the human heart, we find several receptors for monoamines that activate adenylyl cyclases (AC). Such receptors include the 5-HT_4_-serotonin-receptors, the β-adrenoceptors as well as H_2_-histamine receptors (Figure 1). These three receptors convey positive inotropic effects in HAP (e.g., [1,2,3]. They have in common 5-HT_4_-serotonin receptors like β-adrenoceptors, but also H_2_-histamine receptors that activate adenylyl cyclases (ACs) via stimulatory GTP-binding proteins [4,5]. This leads to an enhanced phosphorylation state of, for example, phospholamban in the sarcoplasmic reticulum. Increased phosphorylation of phospholamban can, in part, explain an increase in force of contraction, in the rate of tension (or force) development, in the rate of tension relaxation, the shortened time to peak tension, and the shortened time to relaxation with isoprenaline, serotonin, and histamine in the heart. The phosphorylation of phospholamban is reversed by PP1 and PP2A [6,7]. Serotonin plays a wide role in cardiac function. For instance, serotonin can be formed in the heart and can exert autocrine and paracrine actions [4,5]. Of note, serotonin can increase the beating rate of the human heart by the stimulation of 5-HT_4_-serotonin receptors in the sinus node. Stimulation of 5-HT_1_- and 5-HT_2_-serotonin receptors can lead to detrimental constriction of the coronary arteries. Stimulation on 5-HT_2B_ receptors on fibroblasts in the heart can lead to defects of valve closure. Thrombi, for instance, in the right atrium, can locally release serotonin, and this released serotonin can lead to supraventricular arrhythmias [3]). Finally, serotonin induces a positive inotropic effect in the isolated muscle strips from the right and left atrium via 5-HT_4_-serotonin receptors [8,9]. The density of 5-HT_4_-serotonin receptors increased in rat models of cardiac hypertrophy and in human heart failure. Plasma levels of serotonin increased in hypertension, sepsis, and heart failure (reviews: [4,10].

Carbachol reduces, in atria, the positive inotropic effects of isoprenaline in many species (mouse: e.g., [11], HAP: e.g., [12]. Not only serotonin, but also isoprenaline and histamine augment the phosphorylation state of phospholamban in HAP [13]. These isoprenaline-induced phosphorylations of cardiac regulatory proteins are reduced, at least in the whole isolated hearts from guinea pigs and from rats or in isolated guinea-pig cardiomyocytes by carbachol (e.g., [14]. After isoprenaline treatment, carbachol reduced the phosphorylation of regulatory proteins in the atrium and ventricle of mammals (e.g., [14]). However, there is controversy regarding how this comes about. Some showed that carbachol reduced isoprenaline-stimulated cAMP levels in isolated mammalian atrium and ventricle. Others, and ourselves, failed to measure such reductions in cAMP. At least in part, isoprenaline reduced the activity of PP1, and additionally, applied carbachol increased PP1 activity in the guinea pig ventricle, which would explain the reduction in the phosphorylation state of phospholamban [15].

Cantharidin inhibited the activity of the catalytic subunits of PP1 and more potently PP2A from the human heart [16,17]. Cantharidin failed to raise the cAMP levels but elevated force of contraction in guinea-pig papillary muscles and also in human atrial and ventricular cardiac preparations [16,18,19]. This increase in force of contraction by cantharidin was accompanied, and therefore, in our view was caused, by increased phosphorylation of cardiac proteins in animal hearts and in the human heart like the L-type calcium ion channel or phospholamban [16,18]. Moreover, another way to inhibit PP1 and PP2A is the use of sodium fluoride. We have shown that sodium fluoride at the concentration used in this study, namely 3 mM, inhibited the activity of PP1 and PP2A from the mammalian heart [17]. Pre-treatment with sodium fluoride could attenuate the negative inotropic effect of carbachol after force had been elevated by isoprenaline in guinea pig or rat ventricular preparations [17]. Likewise, sodium fluoride inhibited the negative inotropic effect of carbachol alone in mouse atrium or HAP [19]. Our current hypothesis is that M_2_-muscarinic cholinoceptors act via the activation of PP in the HAP. We assume that carbachol acts in the mouse atrium and the human atrium via M_2_-muscarinic receptors. This view results from, firstly, the fact that in M_2_-KO mice, carbachol cannot reduce force of contraction or beating rate [20]. Secondly, in the human atrium, the negative inotropic effect of carbachol is attenuated by M_2_-receptor antagonists [21,22]. It has not been previously reported whether or not carbachol reduced the serotonin-stimulated force of contraction in left atrial preparations from 5-HT_4_-TG, which we studied here for comparison. Moreover, for comparison, we also studied whether carbachol could reduce the force of contraction in H_2_-TG, which is likewise a novel hypothesis.

Therefore, we studied the main hypothesis:

That cantharidin attenuates the negative inotropic effect of carbachol in the presence of serotonin in left atrial preparations from 5-HT_4_-TG and HAP.

## 2. Materials and Methods

### 2.1. Generation of Transgenic Mice

We have previously described the generation of 5-HT_4_-TG and H_2_-TG [13,23]: basically, we cloned the human H_2_-histamine receptor or the human 5-HT_4_ serotonin receptor into a cloning site of a construct that harbored the α-myosin heavy chain promoter. In this way, one can ascertain that the receptor of interest is expressed in the atrium of the adult mouse heart. The genes are inherited in a heterozygous fashion. In this way, each litter contains wild type and transgenic animals of both sexes to about identical numbers.

### 2.2. Contractile Studies in Mice

Handling, keeping, and sacrifice of mice followed the current European guidelines and had a permit from the local regulators (Veterinäramt der Stadt Halle: I8M9). We used animals of random sex. The mice were aged about 186 ± 24 days. The animals were sacrificed by cervical dislocation. The heart was rapidly excised and placed into a buffer-filled Petri dish under a dissecting microscope. Left atrial preparations from wild type mice or H_2_-TG or 5-HT_4_-TG were isolated and mounted in organ baths of 10 mL organ bath volume [11,13]. The bathing solution of the double-jacketed organ baths and the Petri dishes was a modified Tyrode’s solution and contained 119.8 mM NaCl, 5.4 mM KCl, 1.8 mM CaCl_2_, 1.05 mM MgCl_2_, 0.42 mM NaH_2_PO_4_, 22.6 mM NaHCO_3_, 0.05 mM Na_2_EDTA, 0.28 mM ascorbic acid, and 5.05 mM glucose. The solution was continuously gassed with 95% O_2_ and 5% CO_2_ in order to maintain a pH 7.4 in the bath and to ascertain sufficient oxygen content in the buffer. Ascorbic acid is included here as our standard procedure because we have found this necessary to impair the rapid oxidation of serotonin or isoprenaline (e.g., [12]. The buffer in the organ baths were kept at 37 °C by the circulation of warm water through the outer jacket of the organ baths from a thermostat pump (Lauda company, Lauda-Königshofen, Germany).

We mounted the left atrial preparations vertically, having attached metal hooks to each end of the atrium. The rod contained two platinum stimulation electrodes. Then atria were stretched under isometric conditions until maximum force was generated. This stretching was monitored via transfer of the force via a thin metal wire from the atria to a force transducer (Hellige, Freiburg, Germany). This signal went into a bridge amplifier (AD instruments): Signals were digitized in a PowerLab (ADInstruments, Oxford, UK) and fed into a commercial personal computer (Dell, Halle, Germany). Signals were quantified for developed force, time of contraction, and rate of contraction using commercial software (LabChart version 8.1.31, ADInstruments). Left atrial preparations did not beat spontaneously but were electrically stimulated with 1 Hz of direct current for 5 milliseconds and at about 3–5 V, just sufficient to initiate muscle contraction. For electrical stimulation, we used a Grass SD 9 stimulator (Quincy, MA, USA). The drug application in mouse left atrial preparations was as follows. After equilibration, 30 µM cantharidin was added to left atrial preparations until a plateau in the generation of force had been reached, then a single concentration of isoprenaline, histamine, or serotonin was applied to each left atrial preparation. Then, where indicated in the figures, carbachol was applied to the preparations.

### 2.3. Contractile Studies on Human Preparations

We measured contraction in HAP with the same equipment and solutions as we used in the mouse atria (cf. preceding section). HAP do not beat spontaneously because they do not contain the sinus node. Thus, HAP were also electrically stimulated (vide supra).The HAP were obtained from male and female patients aged between 57 and 85 years. Cardiac comorbidities included atrial fibrillation, coronary heart disease, heart failure, and hypertension. Cardiac drug therapy included metoprolol or a similar β-adrenoceptor antagonist, furosemide or a similar diuretic drug, apixaban or a similar anticoagulant drug, statins, and acetyl salicylic acid. Non-cardiac morbidities of the patients were: anemia due to iron deficiency, bladder cancer, epilepsy, gastritis, hyperthyreosis, hyperlipidemia, hyperuricemia, obesity, prostatomegaly, renal insufficiency, shingles, and type 2 diabetes. Our methods used for atrial contraction studies in human samples have been previously published [16,17,18].

The drug application in left atrial preparations was basically as follows. After equilibration, 100 µM cantharidin was added to HAP until the increase in force had reached a new plateau; then a single concentration of isoprenaline, histamine or serotonin was applied to each HAP. Then, where indicated, carbachol was applied to the HAP.

### 2.4. Data Analysis

Data shown in the figures are arithmetic mean values ± standard error of the mean. We first tested the data for normality with the Shapiro–Wilk test with GraphPad Prism 9. Thereafter, statistical significance was evaluated using ANOVA and Bonferroni’s multiple comparisons test or the Student’s *t*-test as appropriate. We arbitrarily defined a *p*-value less than 0.05 as significant.

### 2.5. Drugs and Materials

Isoprenaline-hydrochloride, histamine dihydrochloride, serotonin hydrochloride, and carbachol were dissolved in water, whereas cantharidin (CANT) was made 100 mM in dimethylsulfoxide (DMSO), and carbachol (CAR, dissolved in water) were purchased from Merck (Darmstadt, Germany). All other chemicals were of the highest purity grade commercially available. Deionized water was used throughout the experiments. Stock solutions were prepared daily.

## 3. Results

### Mouse Left Atrium

**Figure 2:**
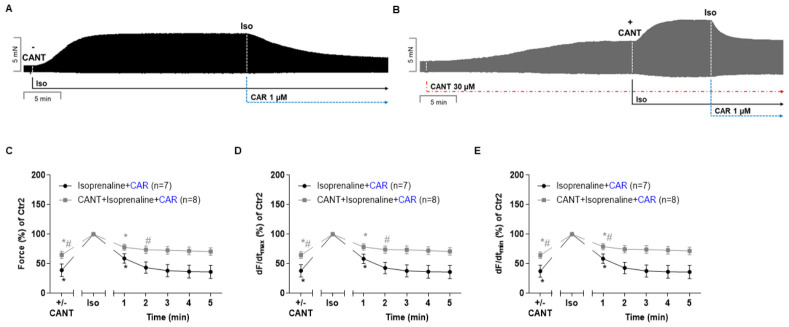
Isoprenaline—Mouse left atrium. Effect of carbachol (CAR) in the presence of isoprenaline (Iso, (**A**)) in the additional presence or absence of cantharidin (+/− CANT, (**B**)) on the force of contraction. Time dependence of the effect of CAR in the presence of isoprenaline alone (Iso = 3.67 ± 0.34 mN, n = 7) or after CANT (Iso = 7.11 ± 1.44 mN, n = 8) on the force of contraction (**C**), the maximum rate of tension development ((**D**), Iso = 141 ± 19.9 mN/s, n = 7; 262 ± 61.5 mN/s, n = 8), or on the maximum rate of tension relaxation ((**E**), Iso = −134 ± 15.9 mN/s, n = 7; −242 ± 54.6 mN/s, n = 8). * and # indicate first significant differences (*p* < 0.05) versus Iso (100%) or versus without CANT, respectively. Numbers in brackets indicate the number of experiments.

Isoprenaline (1 µM) alone augmented contraction in the left atria from wild type mice (Figure 2A). Additionally, the applied 1 µM of carbachol reduced the force of contraction (Figure 2A). This finding and these concentrations are in agreement with the published reports in mouse left atrial preparations from our group under these experimental conditions (e.g., [11]). In a separate preparation, cantharidin by itself exerted a positive inotropic effect in left atrial preparations from wild type mice (original tracings in Figure 2B). Please note that we utilized 30 µM cantharidin in mouse atria and 100 µM cantharidin in HAP (as we have previously reported: [18,19] (original tracings in Figure 2B). The positive inotropic effect of cantharidin was further augmented by additional isoprenaline (original tracings in Figure 2B). Thereafter, 1 µM carbachol was added to the organ bath (original tracings in Figure 2B). Please consider that as in Figure 2A and also in Figure 2B, 1 µM of carbachol exerted a negative inotropic effect. However, in the presence of cantharidin, the negative inotropic effect of 1 µM carbachol in the additional presence of isoprenaline was attenuated in extent and time (cf. original recordings in Figure 2A with Figure 2B). These data over time were summarized from several experiments. Data were compared with and without 30 µM cantharidin for developed force of contraction (Figure 2C). Likewise, in these atria, carbachol reduced the isoprenaline-stimulated rate of tension development (Figure 2D) and the isoprenaline-stimulated rate of tension relaxation (Figure 2E). We have expressed here and in subsequent figures the rate of tension development and the rate of relaxation in percent of control, as others have also chosen to do in mouse atrial preparations. These effects of carbachol were more pronounced in the absence than in the presence of cantharidin.

**Figure 3:**
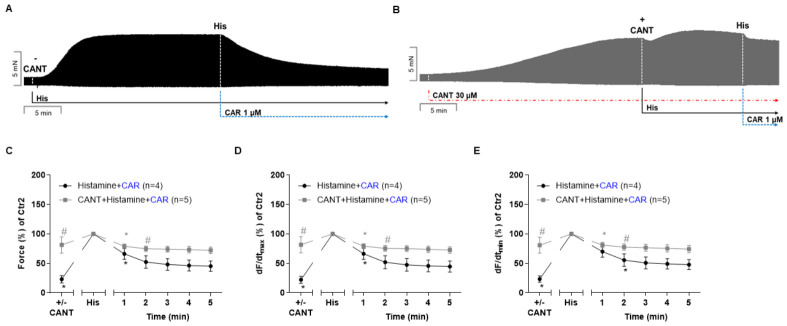
Mouse left atrium from H2-TG. Effect of carbachol (CAR) in the presence of histamine (His, (**A**)) or in the additional presence or absence of cantharidin (+/− CANT, (**B**)) on the force of contraction. Time dependence of the effect of CAR in the presence of histamine alone (His = 5.37 ± 0.80 mN, n = 4) or after CANT (His = 6.88 ± 0.46 mN, n = 5) on the force of contraction (**C**), the maximum rate of tension development ((**D**), His = 283 ± 83.4 mN/s, n = 4; 327 ± 52.0 mN/s, n = 5), or on the maximum rate of tension relaxation ((**E**), His = −204 ± 25.5 mN/s, n = 4; −253 ± 15.7 ms, n = 5). * and # indicate first significant differences (*p* < 0.05) versus His (100%) or versus without CANT, respectively. Numbers in brackets indicate the number of experiments.

Next, we interrogated the interaction of histamine and carbachol in a mouse model (Figure 3A). We have repeatedly shown that in left atrial preparations of WT, there exists no positive inotropic effect to histamine (e.g., [23]). Therefore, we generated and now used here mice that express these human H_2_-histamine receptors only in the cardiomyocytes. We have shown that in isolated left atrial preparations of H_2_-TG, histamine exerts a positive inotropic effect [23]. As a result, 1 µM of histamine was able to maximally increase the force of contraction, and so this concentration was used in the present study [23]. Cantharidin increased the force of contraction and force was further elevated by later applied histamine (Figure 3B). Additional carbachol (1 µM) reduced the force of contraction (Figure 3B). However, with cantharidin, the negative inotropic effect of carbachol in the continuous presence of histamine (1 µM) was attenuated (Figure 3B). These data on force of contraction are summarized in Figure 3C. Likewise, under these conditions, the reductions by carbachol in the histamine-stimulated rate of tension development (Figure 3D) and the histamine-stimulated rate of relaxation (Figure 3E) were lessened with cantharidin in the additional presence of histamine.

**Figure 4:**
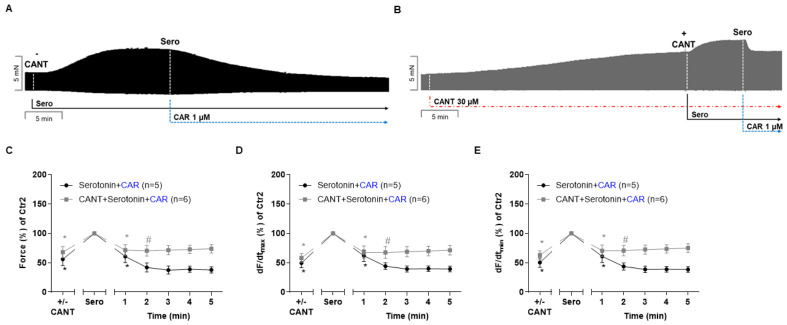
Mouse left atrium from 5-HT4-TG. Effect of carbachol (CAR) in the presence of serotonin (Sero, (**A**)) or in the additional presence or absence of cantharidin (+/− CANT, (**B**)) on force of contraction. Time dependence of the effect of CAR in the presence of serotonin alone (Sero = 3.13 ± 1.04 mN, n = 5) or after CANT (Sero = 5.98 ± 1.07 mN, n = 6) on force of contraction (**C**), on the maximum rate of tension development ((**D**), Sero = 116 ± 31.3 mN/s, n = 5; 364 ± 86.6 mN/s, n = 6), or on the maximum rate of tension relaxation ((**E**), Sero = −106 ± 30.7 mN/s, n = 5; −214 ± 35.1 mN/s, n = 6). * and # indicate first significant differences (*p* < 0.05) Sero (100%) or versus without CANT, respectively. Numbers in brackets indicate the number of experiments.

Subsequently, we wanted to study the interaction of carbachol and serotonin in another transgenic mouse model (Figure 4A) with the aim of contrasting these data with those in HAP (vide intra). Again, we have repeatedly shown that in left atrial preparations of wild type mice, serotonin does not exert positive inotropic effects (e.g., [13]). Therefore, we generated and used here mice that express these human 5-HT_4_ serotonin receptors in the heart. We have shown that in the 5-HT_4_-TG, serotonin augments the force of contraction [23]. Therefore, we used this model, 5-HT_4_-TG, here. As shown above for isoprenaline or histamine, serotonin itself in 5-HT_4_-TG is a lone force of contraction, and this effect was diminished by 1 µM carbachol (Figure 4A). We have previously shown that 1 µM serotonin is effective to stimulate force maximally in this model [13]. In separate experiments, cantharidin in 5-HT_4_-TG increased the force of contraction, which was further increased by 1 µM serotonin. Additionally, application of 1 µM carbachol elicited a negative inotropic effect (Figure 4B). However, in the presence of cantharidin, the negative inotropic effect of carbachol was attenuated (cf. Figure 4A,B). Time-dependent effects are summarized for force of contraction in mN in Figure 4C. Likewise, the reductions by carbachol in the serotonin-stimulated rate of tension development (Figure 4D) and serotonin-stimulated rate of tension relaxation (Figure 4E) were diminished in the presence of cantharidin.

**Figure 5:**
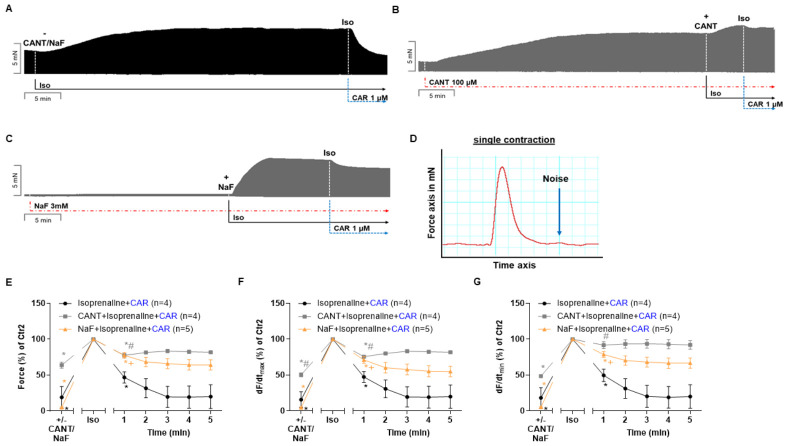
Isoprenaline—Human right atrium. Effect of 1 µM CAR in the presence of isoprenaline (Iso, (**A**)) or in additional presence of CANT (**B**) or NaF (**C**) on force of contraction (CANT/NaF). Time dependence effect of CAR in the presence of isoprenaline alone (Iso = 11.8 ± 1.23 mN, n = 4) or after cantharidin (Iso = 22.4 ± 1.75 mN, n = 4) or NaF (Iso = 13.0 ± 0.83 mN, n = 5) on force of contraction (**E**), on the maximum rate of tension development ((**F**), Iso = 260 ± 34.5 mN/s, n = 4; 614 ± 30.2 mN/s, n = 4; 226 ± 14.1 mN/s, n = 5), or rate of tension relaxation ((**G**), Iso = −140 ± 21.3 mN/s, n = 4; −230 ± 14.5 mN/s, n = 4; −136 ± 6.33 mN/s, n = 5). Representative single contraction in human atrium (**D**). * first significant difference versus Iso (100%). # or + first significance difference versus without CANT or without NaF. Number in brackets indicates the number of experiments.

After these studies in mice, we switched to our main tissue of interest, namely HAP. We used HAP from the right atrium instead from the left atrium, because only samples from the right atrium are routinely obtained in bypass surgery. Again, a single concentration of cantharidin [18] and isoprenaline were utilized (original tracings in Figure 5A,B). We used a higher concentration of cantharidin in human atrium than in mice (100 µM versus 30 µM) because more cantharidin is required to begin to increase the force of contraction in human atrium than in mouse atrium. We have observed this before and have suggested that this can be explained by a higher basal phosphatase activity in the human heart compared to the mouse heart [19]). We have also previously shown that 100 µL of the solvent DMSO brought about a transient negative inotropic effect (in an organ bath volume of 10 mL: [18]. However, here, we used 10 µL DMSO as a solvent control, which did not raise the force of contraction. Our current stock solution contains 100 mM cantharidin in DMSO. Cantharidin (100 µM) had a positive inotropic effect, which was enlarged by additionally applied isoprenaline (original tracings in Figure 5B). As a control, only isoprenaline was given (Figure 5A). For comparison, we studied the interaction of isoprenaline and carbachol in the presence of another phosphatase inhibitor, namely sodium fluoride. Thereafter, under all conditions, the samples received carbachol (Figure 5C). Carbachol elicited a pronounced negative inotropic effect in all conditions. However, in the presence of cantharidin (Figure 5B) or sodium fluoride (Figure 5C) and isoprenaline, the negative inotropic effects of carbachol at 1 µM was attenuated compared to isoprenaline alone (original recordings in Figure 5A–C). These data are summarized for the negative inotropic effects in Figure 5D. Under these conditions, the reductions by carbachol in the rate of tension development (Figure 5E) and the rate of relaxation (Figure 5F) were reduced by both cantharidin and sodium fluoride.

**Figure 6:**
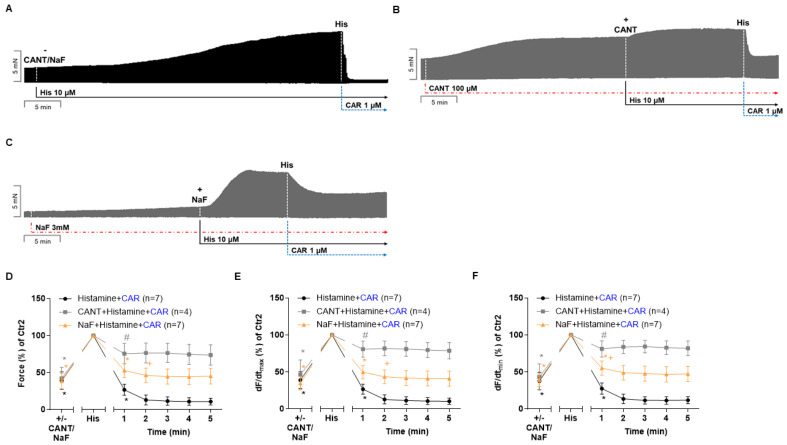
Histamine—Human right atrium. Effect of 1 µM CAR in the presence of histamine (His, (**A**)) or in additional presence of CANT (**B**) or NaF (**C**) on force of contraction (CANT/NaF). Time dependence effect of CAR in the presence of histamine alone (His = 5.26 ± 1.51 mN, n = 7) or after cantharidin (His = 5.48 ± 1.61 mN, n = 4) or NaF (His = 6.16 ± 1.38 mN, n = 7) on force of contraction (**D**), on the maximum rate of tension development ((**E**), His = 118 ± 34.9 mN/s, n = 7; 120 ± 37.7 mN/s, n = 4; 95.1 ± 20.9 mN/s, n = 7), or rate of tension relaxation ((**F**), His = −54.6 ± 11.2 mN/s, n = 7; −64.8 ± 15.1 mN/s, n = 4; −52.1 ± 11.6 mN/s, n = 7). * first significant difference versus His (100%). # or + first significance difference versus without CANT or without NaF. Number in brackets indicates the number of experiments.

Furthermore, the same protocol for histamine as in mice was also employed in human atrial preparations. Again, a single concentration of cantharidin (100 µM) or sodium fluoride (3 mM) exerted a time-dependent positive inotropic effect. Histamine alone (Figure 6A), with cantharidin (Figure 6B) and sodium fluoride (Figure 6C), increased the force of contraction. Thereafter, carbachol was applied. A total of 1 µM carbachol elicited a pronounced negative inotropic effect, which was quantified for force of contraction in Figure 6D. However, with cantharidin and sodium fluoride, the negative inotropic effects of carbachol at 1 µM was lessened (Figure 6D). Under these conditions, the reductions by carbachol in the histamine-stimulated rate of tension development (Figure 6E) and histamine-stimulated rate of relaxation (Figure 6F) were weakened by both cantharidin and sodium fluoride.

**Figure 7:**
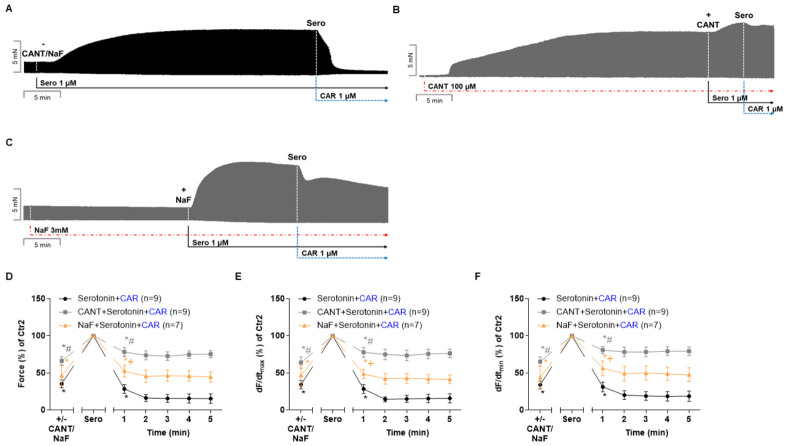
Serotonin—Human right atrium. Effect of 1 µM CAR in the presence of serotonin (Sero, (**A**)) or in additional presence of CANT (**B**) or NaF (**C**) on force of contraction (CANT/NaF). Time dependence effect of CAR in the presence of serotonin alone (Sero = 4.60 ± 1.39 mN, n = 9) or after cantharidin (Sero = 4.07 ± 0.64 mN, n = 9) or NaF (Sero = 8.56 ± 1.72 mN, n = 7) on force of contraction (**D**), on the maximum rate of tension development ((**E**), Sero = 99.2 ± 35.6 mN/s, n = 9; 100 ± 18.2 mN/s, n = 9; 158 ± 36.1 mN/s, n = 7), or rate of tension relaxation ((**F**), Sero = −44.0 ± 10.6 mN/s, n = 9; −60.2 ± 8.73 mN/s, n = 9; −75.2 ± 15.3 mN/s, n = 7). * first significant difference versus Sero (100%). # or + first significance difference versus without CANT or without NaF. Number in brackets indicates the number of experiments.

Finally, again, a similar protocol for serotonin was utilized in the human atrial preparations as in mice. A single concentration of cantharidin (100 µM) exerted a time-dependent positive inotropic effect. Serotonin alone (Figure 7A) or serotonin with cantharidin (Figure 7B) or 3 mM sodium fluoride (Figure 7C) increased the force of contraction further. We used 100 µM cantharidin because lower concentrations of cantharidin did not increase the force of contraction in human atrial preparations [18]. Thereafter, carbachol was applied. A total of 1 µM of carbachol exerted a negative inotropic effect (Figure 7A–C). These effects for force of contraction were quantified from several experiments in Figure 7D. However, in the presence of cantharidin or sodium fluoride, the negative inotropic effects of 1 µM carbachol were diminished (Figure 7D). Under these conditions, the reductions by carbachol in the serotonin-stimulated rate of tension development (Figure 7E) and the serotonin-stimulated rate of relaxation (Figure 7F) were lessened by both cantharidin and sodium fluoride.

## 4. Discussion

In our eyes, the most notable finding in this report lies in the observation that cantharidin and sodium fluoride attenuated the anti-β-adrenergic, anti-serotoninergic, and anti-histaminergic negative inotropic effects of carbachol in the HAP.

It is known that HAP respond to isoprenaline, histamine, and serotonin with an increase in force via their respective receptors (Figure 1). In other words, in the human heart, more specifically in the isolated human atrium, the β-adrenoceptor, the H_2_-histamine receptor, and the 5-HT_4_-serotonin receptor are known to mediate positive inotropic effects.

Carbachol (or acetylcholine) reduced the positive inotropic effect of isoprenaline or histamine or serotonin in the human atrium in the organ bath (e.g., [2,9,12]. The positive inotropic effects of serotonin, histamine, and isoprenaline were attenuated by carbachol, and this effect of carbachol was attenuated by cantharidin, but also to a smaller extent by sodium fluoride in the human atrium in the organ bath.

Carbachol reduced the positive inotropic effects of isoprenaline in the wild type mouse left atrium in the organ bath (e.g., [11]. However, novel findings are that carbachol reduced the positive inotropic effect of serotonin in left atrial preparations from 5-HT_4_-TG and that of histamine in H_2_-TG: these negative inotropic effects of carbachol were attenuated by cantharidin.

Positive inotropic effects of isoprenaline in the guinea pig ventricle involve an increase in the phosphorylation state of phospholamban. This increase in the phosphorylation of phospholamban is probably augmented by an increased phosphorylation state of the phosphatase inhibitor 1 ([24] isolated guinea pig hearts). This phosphorylation in turn leads to activation of the phosphatase inhibitor 1, which then inhibits the enzymatic activity of PP1 [15]. Carbachol increased the PP1 activity but only when it had been previously reduced by isoprenaline [15]. Moreover, activation by carbachol is not necessarily confined to the activation of PP1. In the isolated perfused rat heart, carbachol mainly activated PP2A [25], but this might be a species difference. The increase in rate of tension development by isoprenaline is mainly dependent on phospholamban and its phosphorylation state [26]. There is evidence for a causal relationship for at least two reasons. The increase in the rate of tension development and the rate of relaxation follows closely in time to the increase in phospholamban phosphorylation [27]. Moreover, when they deleted phospholamban from the mouse heart, isoprenaline was less effective to raise the force of contraction and less effective to increase the rate of tension development and the rate of relaxation [28]. We routinely compared the effects of drugs on force with data on the first derivative of this force versus time (dF/dt, cf. original recording in Figure 5D), which was conducted for two reasons. Firstly, to facilitate a comparison with our own previous work (e.g., [29,30]). Secondly, because the first derivatives are more sensitive markers on the intrinsic contractility of a muscle than force or in animals or patients. This has been studied intensively by others (e.g., [31]), who noted that isoprenaline was more potent in raising the rate of tension development (+dF/dt) and suggested that +dF/dt is a better measure of intrinsic contractility of the mammalian heart than the developed force of contraction [31]. Moreover, in percent, isoprenaline increased contractility more effectively; when the first derivative of left ventricular pressure was studied, they noted larger effects that when measuring the left ventricular systolic pressure [31]).

In the present study, the effect of isoprenaline on force and its first derivative were similar when expressed in percentage. The first derivative, absolute numbers in mN/s, is always larger than the force values in mN. In addition, the absolute value for +dF/dt is about double the value of −dF/dt. For instance, after isoprenaline in HAP (Figure 5), we calculated 11.8 ± 1.23 mN. Under these conditions, we calculated +dF/dt 260 ± 34.5 mN/s and −dF/dt as −140 ± 21.3 mN/s. In other words, the atria of mice and humans contract with a higher speed than they relax, which is well-known. The new information we can provide here is that the speed at which tension mounts and falls is similarly affected percentage-wise as in the absolute force values by carbachol. Both contractile rate parameters (rate of tension, rate of relaxation) were similarly less attenuated by carbachol, if cantharidin was present. Moreover, different physiological and biochemical processes determine receptor mediated increases (with isoprenaline) or decreases (with addition carbachol) in force of contraction. For instance, genetic studies (overexpression and knock out of phospholamban or troponin I) suggest that mainly phospholamban and also troponin I and myosin-binding protein C underlie the relaxant properties in mouse atria [28,32,33,34]. One could speculate that the same holds true for the human atrium. The rate of tension development is mainly determined by phospholamban, the ryanodine in the junctional sarcoplasmic reticulum and the L-type calcium cation channel in the sarcolemma [4,17,28,34]. Hence, phospholamban is in part responsible for the speed of tension and speed of relaxation, but other proteins certainly also come into play. This means that our data are hypothesis-generating and can motivate us to study the phosphorylation and function of the four mentioned regulatory proteins in subsequent work. Our data have further merit because we did not only raise the force and speed in the human atrium by using isoprenaline, instead, we show that there seems to be a general phenomenon because we observed similar results with serotonin and histamine, which do not signal via the β-adrenoceptor. Instead, they signal via 5-HT_4_ serotonin receptors and H_2_-histamine receptors, respectively. All three mentioned receptors (Figure 1), belong to the same family of type A G-protein coupled receptors and can increase the cAMP levels in the mouse and human heart via the activation of stimulatory G-proteins and subsequent activation of the adenylyl cyclase in the sarcolemma. When we increased the force of contraction in the atrial preparations of humans or wild type mice (for isoprenaline) or transgenic mice (5-HT_4_-TG: serotonin, H_2_-TG: histamine), the effects of carbachol were attenuated by cantharidin and sodium fluoride, respectively. These new findings indirectly suggest but do not prove that not only the β-adrenoceptor, but also the H_2_-histamine receptors and 5-HT_4_-serotonin receptor in the mammalian atria can activate serine/threonine protein phosphatases (PP1 and/or PP2A). We have presented evidence in the past that this holds true for the interaction of isoprenaline and carbachol with respect to the current through the l-type calcium ion current (LTCC): isoprenaline increased this current in isolated ventricular cardiomyocytes from guinea pigs. This current was reduced by carbachol. This effect of carbachol on the current was attenuated by sodium fluoride, okadaic acid, and cantharidin [35]. These findings suggested to us, but do not prove, that in principle, carbachol in the mammalian cardiomyocytes can activate phosphatases, at least those phosphatases that reduce the activity of the LTCC. Based on these findings, we suggest, but cannot prove from the present data, that carbachol may also activate phosphatases in the mouse and human atrium. We hypothesize that the M_2_-muscarinic receptors activate PP1 and/or PP2A. This activation is most likely crucially mediated by inhibitory G-proteins, conceivably by indirectly reducing the catalytic activity of PP1/or PP2A in the heart. We arrived at this hypothesis from the observation that in atria from animals treated with pertussis toxin (which inactivates inhibitory G-proteins by covalent modification), the negative inotropic effects of carbachol in the presence of isoprenaline are nearly abolished [11].

Human atria have the advantage that they are certainly of clinical relevance. Human atria have the disadvantage that all patients usually suffered from more than one disease, which may have altered signal transduction in their atrium. Therefore, we generated and studied transgenic mice for the human 5-HT_4_ serotonin receptor and the H_2_-histamine receptor. The mice were disease-free and drug-free. Qualitatively, we obtained the same findings in the mouse atria as in the human atria. Therefore, in our view, the experiments in transgenic mouse atria strongly support our findings in human atria. Admittedly, mouse cardiac pharmacology is not identical to human cardiac pharmacology, but currently, this has to be accepted as a limitation of our studies.

Similarly to the findings described above for isoprenaline, we have reported in H_2_-TG, that histamine increased phosphorylation of the state of phosphatase inhibitor 1 [23]. Thereafter, carbachol might stimulate PP1 and/or PP2A, previously inhibited by isoprenaline, serotonin or histamine, but this is speculative. We assume that this activation of PP by carbachol in HAP was impaired by cantharidin or sodium fluoride. The remaining negative inotropic effects of carbachol in the permanent presence of cantharidin might be due to the activation of potassium ion channels (but see [36] for an opposite view) or the inhibition of LTCC or reduction in cAMP or increase in cGMP in the atrium (review: [21]). Moreover, one can argue that cantharidin is less potent or selective than okadaic acid to inhibit PP1 and PP2A. However, okadaic acid is prohibitively costly for use in organ baths (having a volume of 10 mL). Instead, we used it to strengthen our point (the involvement of phosphatase in the negative inotropic effect of carbachol in the presence of e.g., serotonin), in addition to cantharidin, another readily available phosphatase inhibitor, namely sodium fluoride. Sodium fluoride, of course, also has additional effects at 3 mM. For example, sodium fluoride stimulated the activity of phospholipase C [37], can inhibit and stimulate the calcium ion pump of the sarcoplasmic reticulum [38], inhibits the sarcolemmal sodium, potassium ATPase [39], and can interact with GTP-binding proteins [37,40,41]. Additionally, cantharidin does not only inhibit phosphatases, but also has additional effects on other targets [42]. However, we argue here that cantharidin and sodium fluoride have in common that they inhibit both PP1 and PP2A. Hence, the fact that both cantharidin and sodium fluoride, while from distinct chemical classes (organic versus inorganic molecules), would both attenuate the effects of carbachol in the presence of e.g., serotonin and histamine, probably strengthens our interpretation that under these circumstances, carbachol acts in part as a phosphatase activator in the human atrium. Others have suggested that the interaction of isoprenaline and carbachol is based on a common antagonistic action on the sodium-calcium exchanger in an animal model [41]. Interestingly, the sodium-calcium exchanger binds to and is dephosphorylated by PP1 and PP2A [43,44]. Other targets for the action of carbachol are potassium channels and LTCC. In human atrial preparations, others have noted that carbachol opened potassium channels, leading to hyperpolarization, shortening of the duration of the action potential, and conceivably as a result of a negative inotropic effect [35,45] although this view has been challenged [34]). However, their findings might be reconciled with our results, because the activity of cardiac potassium channels or LTCC can be regulated by cardiac phosphatases [19,20,46,47,48]. Indeed, cardiac potassium channels and LTCC can be dephosphorylated by PP1 and PP2A, and that this dephosphorylation can alter their function in the heart [47,48]).

Limitations of the study: We currently do not have access to human ventricular muscle strips. Therefore, we do not know whether similar results would be obtained in the human ventricle as in the human atrium, as in the present study. Cantharidin and sodium fluoride have the drawback in that they act in all cells, and not only within cardiomyocytes. Hence, we cannot exclude that cantharidin and sodium fluoride might have acted in the present study, at least in part, through other cells in the heart. Moreover, we did not provide data on how carbachol in the presence of serotonin could increase the activity of phosphatases. This is due to the limited tissue available. Others have succeeded in reporting that carbachol augmented phosphatase activity that had been reduced by isoprenaline in isolated perfused guinea pig hearts ([15]). Their assay found an increase in the activity of PP1 only in cardiac membranes that also contained sarcolemma. The hearts in their study weighed about one gram. In contrast, the atrial preparations that we used here in the human or mouse atrial preparations weighed only about 4–6 milligrams. We have not yet succeeded in preparing enough yield of membranes to measure phosphatase activity in each frozen atrium. Moreover, we have not yet measured the protein phosphorylation under our experimental conditions to better study the biochemical targets involved. Such studies are hampered by the paucity of human atria available to us. Hence, such study of protein phosphorylation in individual human atrial muscle strips was beyond the aim of the present study. In addition, we only used single concentrations of receptor agonists, namely 1 µM serotonin, 10 µM histamine, and 100 µM cantharidin, in our contraction studies in HAP. This was conducted because these concentrations of serotonin or histamine have been shown to maximally stimulate the force of contraction under our experimental conditions [16,17,18]. We used 100 µM cantharidin here because higher concentrations damaged the muscle leading to contracture in the HAP. Lower concentrations like 30 µM cantharidin hardly raised the force of contraction in HAP [18]). We did not measure Ca^2+^-handling in HAP. This would require the availability of Ca^2+^-tolerant cardiomyocytes prepared from the human atrium; a method that we have not yet established in our lab. In such cells, it would be possible to study the interaction of isoprenaline and carbachol on intracellular Ca^2+^ transients in a direct fashion. Such experiments are awaited with interest. Our study involved diseased human hearts and not non-failing human hearts. Our study patients suffered from cardiac diseases like coronary heart disease or atrial fibrillation or hypertension and were treated with a β-adrenoceptor antagonist. Cardiac comorbidities can reduce inotropic effects, for instance, of isoprenaline in terminal heart failure [49]). In contrast, the positive inotropic effects in HAP of a stimulation of H_2_-histamine receptors or of cantharidin were not diminished in patients with severe heart failure [50,51]. Chronic treatment of patients with coronary heart disease with a β-adrenoceptor antagonist increases not only the potency of histamine and serotonin, but also isoprenaline to elicit a positive inotropic effect in HAP [9]. Finally, the effectivity of M_2_-muscarinic receptors may be compromised in cardiac diseases like heart failure (electrophysiological effects in canine atrial cardiomyocytes: [52]. Likewise, in isolated human atrial cardiomyocytes, the ability of M_2_-muscarinic receptors to open the acetylcholine-gated inward-rectifier K^+^-current was altered in persistent atrial fibrillation [53]. We would argue that this underscores the usefulness of our transgenic mouse models: the mice were not drug-treated before the contraction studies and did not suffer from arrhythmias, coronary heart disease, or heart failure. Because we obtained similar findings in patient samples and in mouse samples, we can argue that our findings in HAP are not likely to be compromised by drug treatment or comorbidities, but show basic pharmacological control mechanisms.

In conclusion, the main new findings of the present study are that cantharidin and sodium fluoride attenuate the negative inotropic effects of carbachol in the presence of serotonin in transgenic mouse atria, and most importantly in HAP. We suggest that stimulation of the M_2_-muscarinic receptor might have activated human atrial PP that had previously been inactivated by cAMP increasing receptors like the β-adrenoceptor, the H_2_-histamine receptor, and the 5-HT_4_-serotonin receptor, which may contribute to the negative inotropic effect of carbachol in the human heart.

## Data Availability

The data from this study are available from the corresponding author upon reasonable request.
